# Abstract of the Proceedings of the Buffalo Medical Association

**Published:** 1857-04

**Authors:** Sanford B. Hunt


ART. V.—*Abstract of the Proceedings of the Buffalo Medical Association.*

TUESDAY EVENING, March 3, 1857.

The association met.

Present—The President, Dr. Eastman, in the chair. Drs. White, Newman, Nichols, Rochester, Whitney, Wilcox, Strong, Dayton, Miner, Hutchins, Hunt, Lemon, Nott, Treat, Hamilton, and Flint.

The minutes of the preceding meeting were read and approved.

*Prof. Rochester* reported a case of acute glossitis. The patient was a servant girl, aged 30 years. Dr. R. was called on the morning of Feb., 14th. The access was very sudden, the girl having retired well the night before. She was now unable to articulate, the tongue very much swollen and everted, the frœnum obliterated, the organ feeling tense, hard and hot to the touch. The glands of the neck were swollen, and there was soreness of the fauces and pharynx. On pressing down the tongue the velum palati could be seen injected. A homœopath had seen her that morning, and pronounced it malignant erysipelas, for which he said he could do nothing.

Dr. R. had the patient removed to his ward at the hospital, and applied ice directly to the tongue. This was painful, but after placing the ice in a gauze bag, she found the application grateful. This was continued for twenty-four hours, at the expiration of which time the swelling had subsided so that the throat could be seen. She could not yet speak. Several leeches were applied, and $\frac{1}{6}$ gr. of tart. antim. given hourly. This was maintained for twenty-four hours. At the end of sixty hours from the attack she could articulate, and after remaining a week in the hospital, she returned to service. No suppuration took place.

Prof. R. also stated that during the month of February, he had seen many cases of the diptheritic pharyngitis described at the last meeting by Prof. Flint. They were accompanied by high fever. One of them was followed two days later by a case of scarlatina in another member of the same family.

Also two cases of croup. In one of them he had prescribed pulv. Doveri, grs. ij., hydrarg ch. mit., gr. j, and succeeded in cauterizing the larynx of the child thoroughly with a solution of nitrate of silver (30 grs. to the ℥.) The relief following this application, was sudden and permanent, and the child went on to a rapid recovery.

In the other case the treatment was nominally the same, but owing to the resistance of the child, he was unable to apply the argentine solution satisfactorily. The child convalesced slowly. He wished to add that he did not see the false membrane in either of these cases, that he was called early and could rationally attribute his success measurably to that fact; but that he had no doubt, from the general character of the symptoms, that the cases were both of them of the membranous form of croup.

*Dr. Treat* incidentally mentioned a case of glossitis, which had been caused by exposure, the man being intoxicated and laying out all night.

*Dr. Strong* inquired if we were possessed of a sufficient knowledge of croup to be able always to diagnosticate between the spasmodic and the membranous forms in the earlier stages.

*Prof. Rochester* spoke of the peculiar hoarseness of the cough, and the extreme suddenness of the attack, it most often occurring in the night, as distinctive of the spasmodic form. In the membranous form the attack was apt to be slow and insidious, the symptoms being less threatening at the outset.

*Dr. Miner* thought that a later diagnostic sign was, so far as his experience reached, unfailing. Spasmodic croup rapidly recovered, and true croup was uniformly fatal. He had never had the good fortune to see a case of membranous croup recover, and thought he should doubt his diagnosis in case of a recovery.

*Dr. Treat* thought that the early diagnostic signs of croup were very uncertain. He had seen cases of true croup as sudden in their access as spasmodic.

*Prof. Rochester*, in reply to Dr. Miner, referred to Dr. Ware's experience in relation to the fatality of membranous croup. It was evidently wrong to suppose that it was necessarily fatal. Dr. Ware had found that under the use of evacuants he lost a large proportion of his patients; that with moist air and anodynes the mortality was much lessened; while, after adding topical treatment to these means, a very good measure of success had been obtained.

*Dr. Miner*, in reply, quoted Prof. Clark, who had said that not one in four cases recovered. For himself he had only seen three cases, but had lost all those. In all of them he had adopted the milder treatment, and had resorted to topical treatment, etc. He used alteratives, also, but death had occurred before they could have taken effect. He thought spasmodic croup very rarely fatal, although it is not improbable that many cases die from over-treatment.

*Prof. White* alluded to the wandering character of the discussion, and wished to recall the original question put by Dr. Strong. He thought that in the great majority of cases the suddenness of access and alarming onset of spasmodic croup would serve to distinguish it from the slow and insidious membranous form. He thought, also, that in membranous croup the respiration was uniformly and constantly hurried, while in spasmodic croup it was more paroxysmal.

*Dr. Nott* suggested that the forms of disease were convertible, that spasmodic croup, when neglected, sometimes terminated in membranous croup, and related cases tending to prove this.

*Prof. White* would suppose that these were membranous cases from the outset.

*Prof. Hunt* mentioned the purely nervous character of the one, and the inflammatory type of the other disease. He had seen cases of spasmodic croup associated with convulsions, an attack of croup occurring at night and at once relieved by a warm bath, to be followed next day by a convulsion. He regarded the croup and the convulsion as different expressions of the same pathological condition.

The discussion here terminated with a general expression to the effect that a distinctive diagnosis was ordinarily sufficiently easy, but that care must be exercised, as it was sometimes doubtful in the early stages.

*Prof. White* referred to what he considered the double error of a distinguished eastern teacher, in running a parallel between croup and puerperal fever, as being both curable by bloodletting. He says that peritonitis, like the redoubtable croup, is soon fatal, but like it again, is readily cured by copious and prompt bleeding. Prof. W. considered this a double error, wrong in its application to either disease.

*Prof. Rochester* mentioned a case of atony of the uterus, occurring in a young and robust woman, which seemed to be cansed simply by the draining off, suddenly, of a large quantity of the amniotic fluid. He inquired if atony was a frequent result of such an accident in robust women?

*Prof. White* answered that it was likely to follow the discharge of the waters when dropsy of the amnion had preëxisted and impaired the muscular power of the uterus. In answer to another inquiry, he stated his opinion that sporadic cases of puerperal fever were most often dependent on some accident of delivery.

*Prof. White* reported an unusually large intra-uterine tumor, which he had recently ligated in the case of a lady in Chautauque county. The tumor was fibrous, with large calcareous deposits occurring with it. The os uteri was dilated and the ligature applied, the tumor being somewhat pediculated. The instrument came away on the second day, and the patient convalesced well. The origin of these tumors is most frequently within the fibres of the uterus, and consequently they do not ordinarily take the pediculated form, but may have large bases which it is impossible to ligate. He had known several cases of this variety, where the tumor had exfoliated and sloughed as the result either of scarification or pregnancy. The vitality of them is so low that a few slight incisions, carried through the mucous membrane and outer layer of the tumor, are sometimes sufficient to cause their exfoliation. He recollected three instances where pregnancy and abortion had caused the destruction of such tumors, and the recovery of the patient.

*Prof. Hamilton* remarked that he was not ready to admit that the vitality of these tumors was so readily disturbed, that fibrous tumors occurring in other localities were usually endowed with considerable vitality. He hardly expected other members to follow the plan which he had adopted, though he thought it would add very much to the value of our transactions, were they, like him, to write out their reports of cases for the association. The quesiion of these tumors was important, and though to-night it had been mentioned only in an incidental way, he wished for a thorough and careful discussion.

The subject of the relative danger of incised and punctured wounds of the uterus having been raised,

*Prof. Hamilton* described a case where a woman accidentally sitting down on some knitting work, one of the knitting needles passed up the vagina, wounding the uterus. A sharp metritis followed, and suppuration took place.

*Prof. Rochester* had a case in charge in which metritis had followed an abortion produced by a knitting needle. As to incised wounds it was evident from the result of many surgical operations on the uterus, that they were attended with little danger when the peritoneum was uninjured.

*The President* inquired as to the prevalence of diptheritic pharyngitis mentioned as epidemic, last month, by Prof. Flint.

*Prof. Flint* had seen about twenty cases during the month, accompanied, by sharp frebrile movement, with slight pharyngitis, occasionally exudation, not much swelling, the patients confined to bed, and considerable debility following it. It attacked all ages, but children suffered mcre than adults.

*Drs. Rochester*, *White* and *Treat*, had also seen cases of this kind.

*Dr. Nott* moved that the chair appoint a committee of three, to prepare a memorial and a sketch of a law regulating the fees of physicians called to attend post-mortem examinations before coroners, with instructions to forward the same to Senator Wadsworth, with a request that he bring it before the Legislature.

Carried, and the Chair appointed Drs. Hunt, Nott, and Dayton, such committee.

*Prof. Flint* gave notice of a motion to amend the by-laws by raising the fee for the annual dues to the sum of three dollars.

Laid on the table for one month under the rule.

*Prof. White* moved to instruct the Trustees to procure larger and more convenient rooms, if in their opinion the funds of the association will warrant the increased expense.

And the association then adjourned.

SANFORD B. HUNT, Secretary.

